# Memantine Protects against Paclitaxel-Induced Cognitive Impairment through Modulation of Neurogenesis and Inflammation in Mice

**DOI:** 10.3390/cancers13164177

**Published:** 2021-08-19

**Authors:** Pi-Shan Sung, Pei-Wen Chen, Chia-Jui Yen, Meng-Ru Shen, Chih-Hung Chen, Kuen-Jer Tsai, Chou-Ching K. Lin

**Affiliations:** 1 Institute of Clinical Medicine, College of Medicine, National Cheng Kung University, Tainan 704, Taiwan; pishansung@gmail.com (P.-S.S.); ivaivagodiva@gmail.com (P.-W.C.); 2 Department of Neurology, National Cheng Kung University Hospital, College of Medicine, National Cheng Kung University, Tainan 704, Taiwan; lchih@mail.ncku.edu.tw; 3 Department of Oncology, National Cheng Kung University Hospital, College of Medicine, National Cheng Kung University, Tainan 704, Taiwan; yencj@mail.ncku.edu.tw; 4 Department of Obstetrics and Gynecology, National Cheng Kung University Hospital, College of Medicine, Tainan 704, Taiwan; mrshen@mail.ncku.edu.tw

**Keywords:** chemotherapy-induced cognitive impairment, paclitaxel, neurogenesis, brain-derived neurotrophic factor, inflammation, neuroinflammation

## Abstract

**Simple Summary:**

Chemotherapy-induced cognitive impairment (CICI) is an increasing awareness due to prolonged survival following cancer chemotherapy in cancer patients. Multiple mechanisms have been proposed for CICI induced by various chemotherapeutic agents; however, hippocampal damage and impaired neurogenesis may be one of the mechanisms. The aim of our study was to investigate the interplay between impaired neurogenesis, inflammation, and the symptoms of CICI, including spatial memory dysfunction and mood alteration, in a paclitaxel-treated mice model. In addition, we demonstrated that memantine may serve as a potential therapeutic agent for paclitaxel-induced CICI by modulating neurogenesis and inflammation. However, the treatment regimen may lead to variations in the treatment efficacy, especially in terms of mood dysfunction. Further translational studies may be developed to evaluate the clinical efficacy of memantine in human CICI studies.

**Abstract:**

Chemotherapy-induced cognitive impairment (CICI) is an adverse side effect of cancer treatment with increasing awareness. Hippocampal damage and related neurocognitive impairment may mediate the development of CICI, in which altered neurogenesis may play a role. In addition, increased inflammation may be related to chemotherapy-induced hippocampal damage. Memantine, an uncompetitive N-methyl-D-aspartate (NMDA) receptor antagonist that may enhance neurogenesis and modulate inflammation, may be useful for treating CICI. To test this hypothesis, paclitaxel was administered to eight-week-old male B6 mice to demonstrate the relationship between CICI and impaired neurogenesis, and then, we evaluated the impact of different memantine regimens on neurogenesis and inflammation in this CICI model. The results demonstrated that both the pretreatment and cotreatment regimens with memantine successfully reversed impaired neurogenesis and spatial memory impairment in behavior tests. The pretreatment regimen unsuccessfully inhibited the expression of peripheral and central TNF-α and IL-1β and did not improve the mood alterations following paclitaxel treatment. However, the cotreatment regimen led to a better modulatory effect on inflammation and restoration of mood disturbance. In conclusion, this study illustrated that impaired neurogenesis is one of the mechanisms of paclitaxel-induced CICI. Memantine may serve as a potential treatment for paclitaxel-induced CICI, but different treatment strategies may lead to variations in the treatment efficacy.

## 1. Introduction

Chemotherapy-induced cognitive impairment (CICI), also known as “chemobrain” or “chemo-fog”, is recognized as a frequent adverse effect of cancer treatment. CICI was reported in 78% of the cross-sectional and 69% of the prospective longitudinal studies between 1995 and 2012 in patients treated for breast cancer [1]. The onset and duration of CICI vary clinically. It may occur transiently or be reversible during chemotherapy or after chemotherapy but has also been reported to persist for months to 5–10 years in 35% of patients in disease-free remission [2]. The long-lasting symptoms of CICI may progress, meaning that CICI may have a potential negative impact on patients’ quality of life [3].

Virtually all categories of chemotherapeutic agents have reported adverse neurological effects for patients. CICI may include the disruption of memory, attention, concentration, processing speed, and executive function and mood alteration, including anxiety and depression [4]. Until now, the definite mechanism of CICI has remained unclear, and various chemotherapeutic agents may lead to clinical symptoms of CICI through different mechanisms. The potential mechanisms reported in previous studies include oxidative stress (doxorubicin, cyclophosphamide, and methotrexate); immune dysregulation (doxorubicin, rituximab, and nivolumab); impaired neurogenesis (nivolumab, doxorubicin, fluorouracil (5-FU), cyclophosphamide, and cisplatin); the alteration of long-term potentiation (paclitaxel and dactinomycin); white matter abnormalities (methotrexate and 5-FU); and cerebrovascular alterations (methotrexate) [3]. Any single chemotherapeutic agent may induce CICI via multiple mechanisms. In addition, the occurrence of CICI related to any given chemotherapeutic agent may also be related to clinical factors, including the total dose, administration route (such as intrathecal), receipt of combination cerebral radiation, interactions with other chemotherapeutic agents or other potential drugs that influence cognitive function, and the presence of cerebral structural lesions [5].

Paclitaxel, an anti-microtubule agent, interferes with cell division, proliferation, and cell group disruption. It has been reported to cause CICI symptoms in clinical and animal models [6,7,8], including impaired hippocampal-dependent spatial memory [9]. Paclitaxel-induced acute or delayed CICI has been reported from 5 h to 12 months after treatment [10,11]. However, the exact mechanism of paclitaxel-induced CICI has not been totally elucidated. Despite poor blood–brain barrier (BBB) penetration, an animal study showed that a dose-dense paclitaxel treatment may cause a paclitaxel concentration in hippocampal tissue that is sevenfold higher than that in the neocortex [9]. Cancer patients treated with chemotherapy, including paclitaxel-treated patients [12], may have more severe hippocampal atrophy than healthy controls [13]. Therefore, there may be a potential link between paclitaxel treatment, hippocampal damage, toxicity, and the spectra of its related neurocognitive and mood disturbances [4].

A previous in vitro study showed that paclitaxel may induce neurotoxicity in neural stem cells [9]. However, in addition to direct drug neurotoxicity, a previous Alzheimer’s disease (AD) model and genome-wide association analyses showed that neurogenesis-related pathways are also associated with changes in the hippocampal volume [14,15]. Neurogenesis, or the creation of new neural cells, has indeed been found to continue throughout adult life in the dentate gyrus of the hippocampus and subventricular zone of the lateral ventricle wall [16]. This process may contribute to the normal function of the adult brain and be induced in response to cerebral diseases for self-repair [17]. Disrupted hippocampal neurogenesis may be implicated in cognitive impairment and mood disorders [18,19]. Neurogenesis may be stimulated through extrinsic modulators, such as metabolic growth factors (e.g., insulin-like growth factor-1 (IGF-1) and brain-derived neurotrophic factor (BDNF)) and behavioral factors, such as an enriched environment or exercise, but may be attenuated by a state of acute or chronic inflammation, aging, or chronic stress [20,21]. A cross-sectional study demonstrated cognitive dysfunction and a reduced hippocampal volume in breast cancer patients who received chemotherapy and had symptoms of CICI. This reduced hippocampal volume was found to be associated with elevated serum inflammatory cytokines, especially tumor necrosis factor-α (TNF-α) and interleukin-6 (IL-6) [12]. Therefore, chemotherapy-induced peripheral inflammation may be associated with the development of hippocampal-dependent cognitive dysfunction and related long-term hippocampal damage. Whether the induction of inflammation connects paclitaxel-induced CICI and hippocampal damage with paclitaxel-induced impaired neurogenesis needs further investigation.

Memantine, an uncompetitive N-methyl-D-aspartate (NMDA) receptor antagonist that has been used as a neuroprotective agent to treat moderate-to-severe AD, may have a potential effect on enhancing neurogenesis [22,23]. In addition, in vivo and in vitro studies have shown that memantine modulates neuroinflammation and cytokine reduction after an intraventricular lipopolysaccharide (LPS) injection [24]. Whether memantine protects against CICI by modulating neurogenesis and neuroinflammation warrants further evaluation.

Therefore, in this study, we aimed to characterize neurogenesis in paclitaxel-induced CICI and to elucidate the molecular mechanism by which paclitaxel induces impaired neurogenesis. More importantly, we aimed to establish potential treatment strategies based on memantine for paclitaxel-induced CICI by modulating neurogenesis and inflammation.

## 2. Materials and Methods

### 2.1. Animal Preparation

Eight-week-old inbred C57BL/66NCrlB1tw mice and outbred C57BL/6NCrlB1tw mice were obtained from Lasco (LASCO, Taipei, Taiwan). All animal experiments were carried out according to a protocol approved by the Committee for Animal Experimentation at National Cheng-Kung University (IACUC-106152 and IACUC-109267). We maintained the animal house temperature at 23 ± 3 °C with a relatively stable humidity of 45–65% and a 12-h light/dark cycle (lights on from 06:00 to 18:00). We minimized animal suffering and reduced the number of animals during all experiments. The animals were transferred to the environment at least 1 h before the start of the experiment.

### 2.2. Pharmacological Treatment of Animals

**Figure 1:**
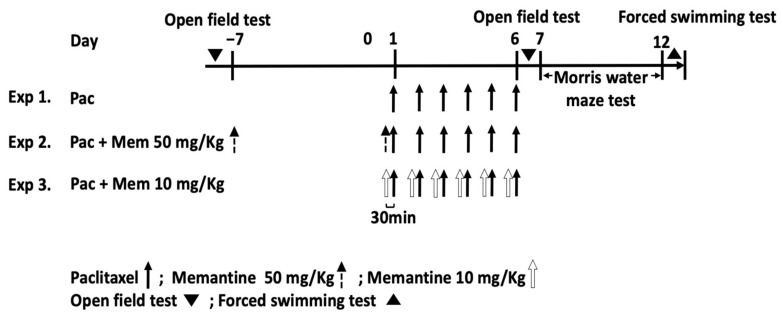
Study protocol for the paclitaxel and memantine treatment. Experiment 1 comprised the control group and paclitaxel group only. Paclitaxel was given intraperitoneally at a dose of 6 mg/kg for 6 consecutive days (Day 1–Day 6). The memantine treatment was separated into 2 regimens (Experiments 2 and 3). The second line is the pretreatment regimen (Experiment 2). Paclitaxel was given intraperitoneally at a dose of 10 mg/kg for 6 consecutive days (Day 1–Day 6). The third line is the cotreatment regimen (Experiment 3). Paclitaxel was given intraperitoneally at a dose of 6 mg/kg for 6 consecutive days (Day 1–Day 6). Memantine was given prior to the paclitaxel or vehicle treatment in Experiments 2 and 3). Exp 1: Experiment 1, Exp 2: Experiment 2, and Exp 3: Experiment 3.

Overall, 3 different sets of animal experiments were performed (Figure 1). (1) In the first set of experiments to demonstrate the behavior/mood changes and to confirm the neurogenesis status in the paclitaxel-induced CICI model (Experiment 1; Exp 1), the mice received the vehicle and paclitaxel each at a dose of 6 mg/kg for 6 consecutive days [25]. Paclitaxel was injected intraperitoneally (i.p.) in appropriate volumes. The placebo group received the vehicle (i.p.). The initial drug toxicity studies were performed at these doses, and no major toxicities were observed (weight loss < 10%). Then, to observe the effect of memantine on the CICI model, the mice were gathered into 4 groups for another two treatments. (2) In the second set of experiments with a pretreatment regimen (Experiment 2; Exp 2), in addition to the control and paclitaxel groups, another two groups of mice were pretreated with high-dose memantine (50 mg/kg; i.p.) one week (on Day−7) prior to and 30 min before (on Day 1) the vehicle or paclitaxel treatment. In the pretreatment regimen, the paclitaxel dosage was 10 mg/kg for 6 consecutive days to further augment the potential pharmacological effect of paclitaxel on the neurogenesis in the CICI model [8]. (3) In the third set of experiments with a cotreatment regimen (Experiment 3; Exp 3), memantine at a dose of 10 mg/kg (i.p.) was given daily concomitantly with a paclitaxel administration for 6 days (Day 1–Day 6). In this regimen, the dose of paclitaxel was 6 mg/kg, which was the same dose used in Experiment 1. Daily memantine was given 30 min prior to the administration of paclitaxel.

### 2.3. Assessment of Learning and Spatial Memory with the Morris Water Maze (MWM) Test

The MWM test was used to evaluate spatial learning and memory. The testing was started after the injection course for 6 days (Day 7–Day 12). The pool was 180 cm in diameter and 50 cm deep. Highly visible cues were positioned on the walls of the tank and around the room. The water temperature was maintained at 25 °C. A clear round platform (10 cm in diameter) was positioned 0.5 cm below the surface of the water and 15 cm from the target region wall. A visible platform trial was performed on Day 1 to ensure that the mice had normal motor functions, with the goal platform raised 1 cm above the water and a clear marker provided. The platform was placed in a region different from the target region. Then, each mouse was subjected to a series of 1 to 2 trials per day. For each trial, the mice were randomized to one of four starting locations (North, South, East, and West) and placed in the pool facing the wall. The maximum time allowed to find the submerged platform was 120 s. If the mouse failed to reach the platform within this time, it was placed on the platform by the experimenter and allowed to remain there for 20 s. The data from each trial was averaged to obtain the final representative latency and the cumulative escape latency of the MWM test on that day.

### 2.4. Assessment of Locomotion and Anxiety with the Open-Field Test (OFT) and of Depressive-like Behavior with the Forced Swimming Test (FST)

The OFT was used to evaluate the locomotor activity and anxiety in the mice. The OFT was performed before and after the complete course of paclitaxel treatment (before Day 0 and after Day 6). In Experiment 2, the first OFT was performed before the first dose of memantine treatment (before Day 7). Before the test, the mice were placed in the test room for 30 min with dim light. The mice were placed individually in the middle of an open-field apparatus placed inside of a larger opaque box (45 cm × 45 cm) with five regions, including the center and the four corners of the area (designated Q1, Q2, Q3, and Q4). The mice were allowed to explore the open field for 5 min, while the horizontal activity reflecting the range of motion was recorded. After each 5-min session, the open-field apparatus was cleaned, and the next mouse was tested. The total distance, time, and distance traveled in the central region of each mouse were recorded by video tracking software (Ethovision XT, Noldus, KYS Technology, Taipei, Taiwan).

The FST was used to evaluate the depressive-like behaviors in Experiments 2 and 3. The FST was performed after the complete course of paclitaxel treatment (after Day 7). During the FST, the mice were placed in an open cylindrical container (diameter 20 cm, height 25 cm) filled with water at 24 ± 1 °C to a depth of 30 cm, and the mice could not escape. The mice were divided into 4 groups: the control, paclitaxel, memantine, and memantine + paclitaxel groups. They were forced to swim for 6 min. The immobility time was measured.

### 2.5. Tissue Preparation and Immunohistochemistry

The mice were sacrificed after behavior testing. The mice were i.p. anesthetized with 10% chloral hydrate (4 mL/kg, Sigma, St. Louis, MO, USA) and then perfused intracardially with PBS. The brains were then removed, postfixed with 4% paraformaldehyde, and cryoprotected in 30% sucrose until they sank to the bottom. We covered the entire tissue block with cryo-embedding media (e.g., OCT) and then stored the frozen tissue block at −80 °C until sectioning. The above-described sections of brain tissue (16 µm) were used for immunofluorescence staining to determine the neurogenesis and neuroinflammation statuses. The markers of neurogenesis, including doublecortin (Dcx), 5-bromo-2′-deoxyuridine (BrdU), and Ki-67, were assessed by the immunohistochemistry staining performed on Day 7 (Dcx) and Days 13 and 14 (BrdU/Ki-67) for 3 different protocols. Immunofluorescence staining for the neuroinflammation marker ionized calcium-binding adaptor molecule-1 (Iba-1) was performed on Day 14. The sections were then incubated overnight with primary antibodies against the following: Dcx (1:200, Cell Signaling, Danvers, MA, USA), Ki-67 (rabbit IgG, 1:100, Abcam, Cambridge, MA, USA), and Iba-1 (mouse IgG, 1:300, Wako, Osaka, Japan). The specimens were incubated with goat anti-rabbit fluorescein isothiocyanate (Rabbit488, 1:300, Life Technologies, Eugene, OR, USA) for 1 h with DAPI. The slides were covered with fluorescence mounting medium (Dako, Santa Clara, CA, USA) and observed under a microscope.

For BrdU (rat IgG, 1:100, Abcam, Cambridge, MA, USA) immunostaining, BrdU (50 mg/kg, i.p.; Sigma, St. Louis, MO, USA) was injected once daily for 6 consecutive days prior to each paclitaxel treatment before mouse sacrifice to evaluate the progenitor cell proliferation. After sacrifice, the brain sections were incubated in 2-N HCl for 60 min to denature the DNA and rinsed with 0.1-M sodium borate buffer to neutralize the acid. After washing in PBS, the sections were incubated with 3% BSA/PBS/0.1% TX-100 overnight at 4 °C. Next, the sections were incubated with secondary Rat488 (Rat488, Life Technologies, Eugene, OR, USA) for 1 h at room temperature. The slides were covered with fluorescence mounting medium (Dako, Santa Clara, CA, USA) and observed under a microscope.

### 2.6. Determination of Cell Number

The number of cells showing the specific characteristics of neuroblasts and proliferating cells (immunopositivity for Dcx, Ki-67, and BrdU) in the hippocampus was scored by an observer blinded to the identity of the sample. The brain from each mouse was sampled at a level of 2.12 mm behind the bregma. The counting of Dcx-, Ki-67-, or BrdU-positive cells in both hemispheres of the dentate gyrus was performed under a microscope. All cells in the subgranular layer of the dentate gyrus from top to bottom were counted.

### 2.7. Determination of Hippocampal Brain-Derived Neurotrophic Factor (BDNF) Expression by Enzyme-Linked Immunosorbent Assay (ELISA)

We evaluated the impact of paclitaxel and memantine on the hippocampal BDNF expression and the associated effects on hippocampal neurogenesis. Four groups with 2 different memantine treatment regimens were assessed: control, paclitaxel, memantine, and memantine + paclitaxel. In the pretreatment regimen (Experiment 2), the BDNF was assessed at 2 time points: 6 h after the last memantine dose and on Day 7 after completing the paclitaxel treatment. In the cotreatment regimen (Experiment 3), the BDNF was assessed on Day 7 after completing the paclitaxel and memantine treatment and Day 14. The mice were sacrificed by decapitation, and the bilateral hippocampi were removed. The hippocampal levels of the BDNF were measured by ELISA. The tissues were homogenized in lysis buffer with a protease inhibitor cocktail. Then, the samples were centrifuged at 9000× *g* for 15 min at 4 °C. The supernatant was then collected, and the BDNF was assessed with a BDNF ELISA kit (R&D Systems, Minneapolis, MN, USA). The data were calculated from the standard curves created with human BDNF for each plate and were expressed as the mean BDNF value (pg) per mg of total protein, which was used to normalize the BDNF results. The results were expressed as concentrations (pg/mL).

### 2.8. Determination of Systemic and Hippocampal Inflammatory Cytokines by ELISA

To determine the effect of memantine treatment on systemic and hippocampal inflammatory cytokine expression, we tested the serum and tissue expression of interleukin-1β (IL-1β) and TNF-α in all the groups. In the pretreatment protocol, serial cytokine expression was assessed at different time points: 6 h after the last memantine dose (Day 1), on Day 7, and on Day 14. In the cotreatment protocol, the cytokines were assessed on Day 7 and Day 14. The serum samples used to assess the cytokine concentrations were isolated from whole blood. The blood sample was allowed to clot for 2 h at room temperature before being centrifuged for 20 min at 2000× *g*. Then, we removed the serum and stored the samples at −20 °C. To determine the tissue expression of inflammatory cytokines, the mice were i.p. anesthetized with 10% chloral hydrate (4 mL/kg, Sigma, St. Louis, MO, USA) and then intracardially perfused with PBS. The brains were then removed and frozen at −20 °C until analysis. The hippocampal tissues were harvested and homogenized. The tissue homogenate protein levels were measured using the BSA method, and the cytokine values were normalized to the protein levels. The levels of the cytokines, including IL-1β and TNF-α, were detected using commercially available ELISA kits (R&D Systems, Minneapolis, MN, USA). ELISA was performed according to the manufacturer’s instructions. The optical density values were measured at wavelengths of 450 nm and 540 nm using a microplate reader (SpectraMax 340PC384, Molecular Devices).

### 2.9. Statistical Analysis

All data were expressed as the mean ± SEM. The data were analyzed using an unpaired *t*-test or one-way analysis of variance (ANOVA), followed by a post hoc Tukey’s test. A data point was defined as an outlier if it was 3 SE above or below the mean of the remaining representative data. Outliers were excluded from the analysis. In all cases, *p* < 0.05 was considered to indicate significance. The data were analyzed by the commercial software SPSS, version 17.0. (IBM Corp., Armonk, NY, USA).

## 3. Results

### 3.1. Paclitaxel Treatment Induced CICI and Impaired Neurogenesis

#### 3.1.1. Paclitaxel Impaired Spatial Learning and Memory Measured by the MWM Test and Increased Anxiety Measured by the OFT

**Figure 2:**
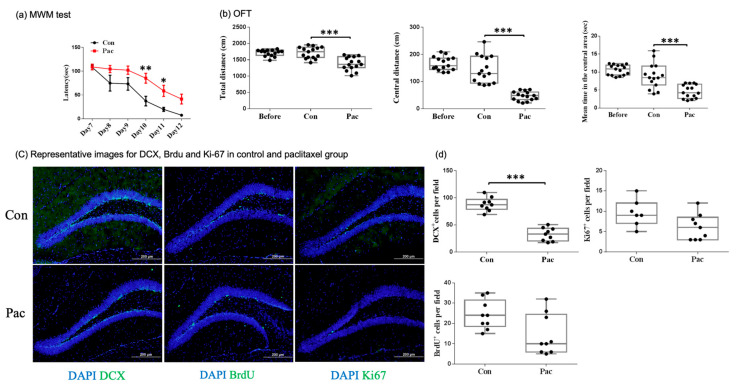
Behavioral tests and neurogenesis assessment after the paclitaxel treatment. (**a**) Paclitaxel impaired spatial learning and memory measured by the MWM test (control group: *N* = 7; paclitaxel group: *N* = 10). (**b**) Paclitaxel impaired locomotor activity and increased anxiety levels measured by the OFT (*N* = 15 in each group). (**c**,**d**) Paclitaxel impaired neurogenesis, especially neuroblast production in the dentate gyrus (*N* = 8 to 9 in each group). The neuroblasts were marked by Dcx immunopositivity. The BrdU- and Ki-67-positive cell counts tended to be reduced, but the differences were not statistically significant (*N* = 8 to 9; *p* < 0.001, *p* = 0.0707, and *p* = 0.1875 for Dcx, BrdU, and Ki-67 staining, respectively). * *p* < 0.05, ** *p* < 0.01, and *** *p* < 0.001.

We first determined the neurocognitive function of the mice in Experiment 1. The paclitaxel treatment significantly increased the duration of the escape latencies compared with the control group (Figure 2a). These data suggested that the acute paclitaxel treatment impaired spatial learning and memory. Then, we determined the locomotor activity and anxiety of the mice that received the paclitaxel treatment by performing the OFT. The total distance traveled, distance traveled, and time spent in the central region were significantly decreased in the group of mice treated with paclitaxel compared with the respective values in the vehicle-treated animals (Figure 2b). These data suggested that the paclitaxel treatment impaired the locomotor activity and increased the anxiety levels in the mice.

#### 3.1.2. Paclitaxel Impaired Hippocampal Neurogenesis

Figure 2c shows representative images of neurogenesis in the dentate gyrus. Dcx was utilized as a marker of newly created neurons or neuroblasts. BrdU was incorporated into newly synthesized DNA during the S phase of the cell cycle and was detectable by immunohistochemistry using specific antibodies against BrdU. Ki-67 immunoreactivity was apparent in the nuclei of the cells located at the border between the granular cell layer (GCL) and hilus. Figure 2d shows the analyzed group data for Dcx, BrdU, and Ki-67 staining after the paclitaxel treatment. The number of cells with Dcx immunoreactivity was significantly decreased in the group of mice treated with paclitaxel compared with that in vehicle-treated animals (*p* < 0.001). The results showed that the number of cells with BrdU immunoreactivity and Ki-67 immunoreactivity tended to be decreased by paclitaxel, but the difference between the groups was not statistically significant. This result indicated that the number of neuroblasts was significantly reduced, and the number of proliferating cells tended to be reduced in the early stage after the paclitaxel treatment.

### 3.2. Memantine Pretreatment Restored Hippocampal Neurogenesis and Improved Memory Deficits but Induced Depression-like Behavior and Maintained Anxiety

#### 3.2.1. High-Dose Memantine Pretreatment Restored Hippocampal Neurogenesis

**Figure 3:**
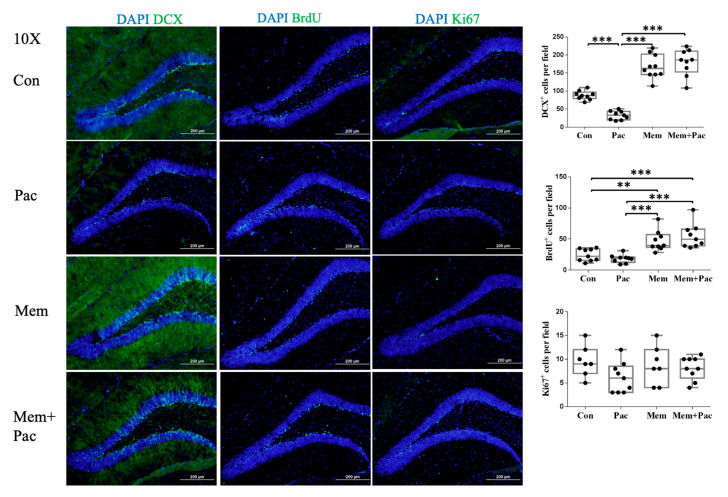
Pretreatment regimen (Experiment 2). Paclitaxel (10 mg/kg of each dose) impaired neurogenesis, especially the neuroblast production in the dentate gyrus (*N* = 7–10 in each group). The neuroblasts are marked as Dcx-immunopositive cells. The BrdU- and Ki-67-immunopositive cell counts tended to be reduced in the paclitaxel-treated mice compared with the control group, but the differences were still not statistically significant. The memantine pretreatment enhanced neurogenesis by increasing the Dcx- and BrdU-immunoreactive cell counts in the memantine group and restored neurogenesis by increasing the Dcx- and BrdU-immunopositive cell counts in the paclitaxel-treated mice pretreated with memantine compared with the paclitaxel treatment with no pretreatment (*p* < 0.001 for both Dcx- and BrdU-immunopositive cells), but the Ki-67-immunopositive cell counts were comparable between the groups. ** *p* < 0.01, and *** *p* < 0.001.

Figure 3 shows representative images and analyzed data of the Dcx-, BrdU-, and Ki-67-immunopositive cells in the four groups. Paclitaxel (10 mg/kg of each dose) impaired the neuroblast production in the dentate gyrus, which was marked as Dcx-immunopositive cells. The BrdU- and Ki-67-immunopositive cell counts tended to be reduced in the paclitaxel-treated mice compared with the control group, but the differences were still not statistically significant. After the memantine pretreatment, the Dcx-immunoreactive cell count was significantly increased in the group of mice treated with memantine compared with the vehicle- and paclitaxel-treated mice (*p* < 0.001). The BrdU-immunopositive cell count was also significantly restored after the memantine treatment compared with that in the paclitaxel group and control group (*p* < 0.001). The Ki-67-immunopositive cells were comparable between the groups.

#### 3.2.2. Memantine Pretreatment Successfully Restored Memory Deficits but Caused Depression-like Behavior and Maintained Anxiety in CICI Mice

**Figure 4:**
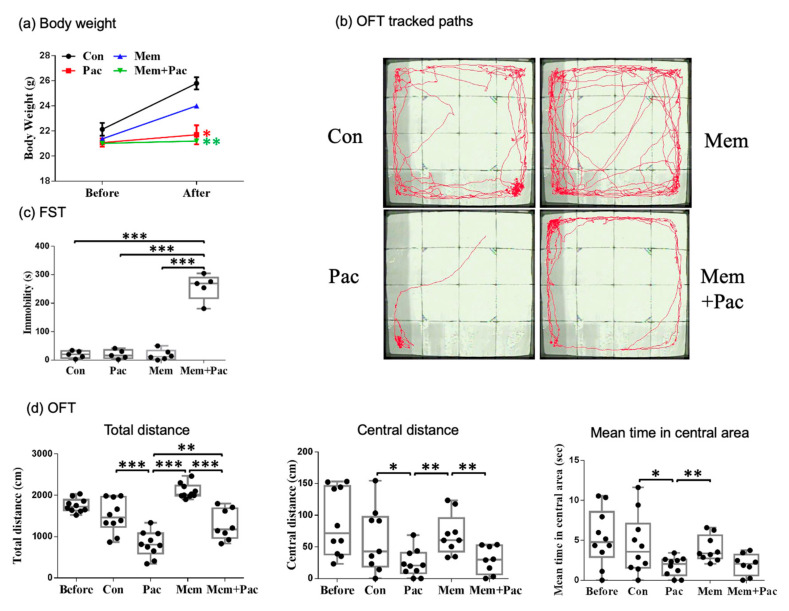
Pretreatment regimen (Experiment 2). (**a**) Body weights of the mice in the four groups after treatment. The data are presented as the mean ± standard error of the mean. The body weights were similar in the paclitaxel and memantine + paclitaxel groups, but both were lower than those in the control group (*N* = 5). (**b**) The OFT tracked paths for the four groups. (**c**) The FST showed an increased immobility time in the memantine + paclitaxel group compared with the other 3 groups (*N* = 5 to 6 in each group). (**d**) Paclitaxel impaired the locomotor activity in both paclitaxel-treated groups, including the memantine + paclitaxel group. The total distance traveled in the memantine + paclitaxel group was still increased compared with that measured in the paclitaxel group (*p* < 0.01). However, memantine + paclitaxel did not reverse the anxiety, as the central distance and mean time in the central area were comparable between the memantine + paclitaxel and paclitaxel groups (*N* = 8–10 in each group). The group in the before column indicates the data of those mice tested with the OFT before any memantine or paclitaxel treatment. * *p* < 0.05, ** *p* < 0.01, and *** *p* < 0.001.

**Figure 5:**
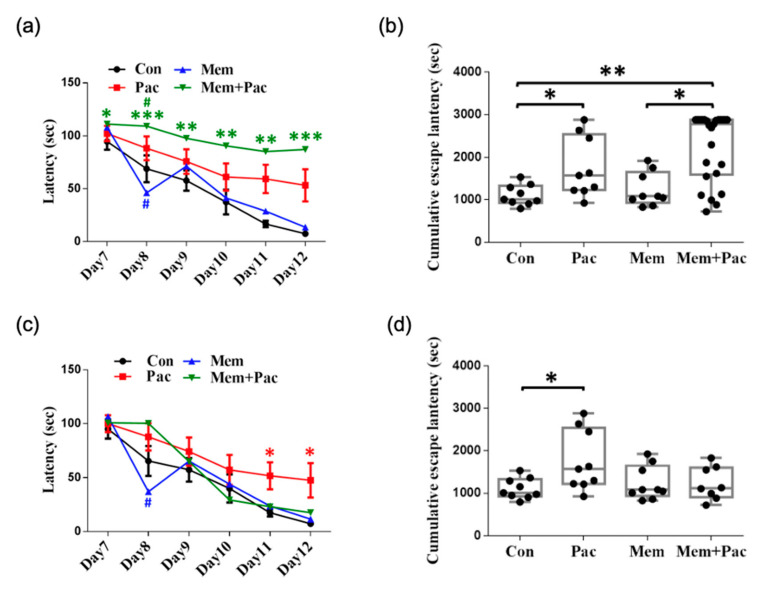
Pretreatment regimen (Experiment 2). (**a**,**b**) The MWM test was performed with mice that received high-dose memantine in the pretreatment regimen (*N* = 9 in the control, paclitaxel, and memantine groups; *N* = 25 in the memantine + paclitaxel group). Memantine + paclitaxel potentially caused immobility of the mice (as measured in the MWM tests). (**c**,**d**) When excluding immobile mice (17/25, 68%), the average escape latency was reduced in the memantine + paclitaxel group to a level comparable to that measured in the control mice. * *p* < 0.05, ** *p* < 0.01, and *** *p* < 0.001 compared with the control group; # *p* < 0.05 compared with the paclitaxel group.

We found that the body weights were similar in the paclitaxel and memantine + paclitaxel groups, but both were lower than those in the control group (Figure 4a). The locomotor activity (measured by the OFT) was comparable between the vehicle-treated and memantine-treated control mice (Figure 4b,d). The results implied that there were no obvious adverse effects related to memantine. The total distance traveled was significantly increased in the memantine + paclitaxel group compared with the paclitaxel group (*p* < 0.01, Figure 4d), but the distance traveled and time spent in the central region were comparable in the memantine + paclitaxel group and the paclitaxel group (Figure 4d). These data suggested that a high-dose memantine pretreatment may not reverse paclitaxel-induced anxiety but may improve the locomotor activity, as demonstrated by the OFT. In addition, the FST further demonstrated an increased immobility time in the memantine + paclitaxel group compared with the other three groups (Figure 4c). The MWM test showed heterogeneity in the memantine + paclitaxel group (Figure 5). The overall escape latencies were significantly increased in the memantine + paclitaxel group compared with the other three groups, including the paclitaxel group (Figure 5a,b). However, 68% of the mice (17/25) did not have any swimming movement (immobility) while performing the MWM test. When excluding these immobile mice from the MWM analysis, the escape latencies were significantly decreased in the memantine + paclitaxel group compared with those measured in the paclitaxel group (Figure 5c,d). These data suggested that a high-dose memantine pretreatment may reverse memory deficits after a paclitaxel treatment but may induce depression-like behaviors, especially in groups receiving memantine + paclitaxel.

#### 3.2.3. High-Dose Memantine Pretreatment Significantly Increased Hippocampal BDNF Levels but Did Not Persistently Inhibit the Elevated Expression of Serum and Hippocampal TNF-α and IL-1β in CICI Mice

**Figure 6:**
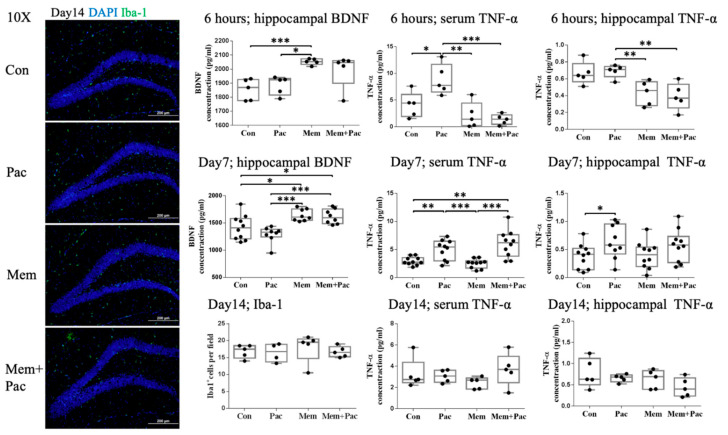
Pretreatment regimen (Experiment 2). The immunostaining for Iba-1 at Day 14 showed comparable microglial activation between the four groups. The changes in the expression of BDNF and TNF-α were different among the four groups. Memantine increased the BDNF expression in the hippocampal tissue after the second high dose of memantine, as demonstrated in the tissue sample extracted 6 h after the second high dose of the memantine treatment. After completion of the paclitaxel treatment, a reduced BDNF expression was noted in the paclitaxel group compared with that in the memantine-treated groups. The memantine treatment increased the BDNF expression in both memantine groups compared with the control group. Memantine suppressed the expression of TNF-α in the serum and hippocampal tissue after the second high dose of memantine. Just after completion of the paclitaxel treatment, an increased TNF-α was noted in both the paclitaxel group and the memantine + paclitaxel group. The TNF-α level became comparable among the groups on Day 14, indicating that the elevation of peripheral cytokines had subsided. Iba-1: *N* = 5 in each group, BDNF: *N* = 5–10 in each group, and TNF-α: *N* = 5–10 in each group. * *p* < 0.05, ** *p* < 0.01, and *** *p* < 0.001.

The ELISA analysis of the BDNF in hippocampal tissue extracts taken on Day 1. 6 h after the first dose of memantine showed that the high-dose memantine pretreatment significantly increased the BDNF in the memantine group (*p* < 0.001, Figure 6) and the memantine + paclitaxel group. At the end of the paclitaxel treatment (Day 7), the BDNF was significantly reduced in the paclitaxel group compared with the memantine and memantine + paclitaxel groups (*p* < 0.001). The memantine treatment also increased the BDNF expression in both memantine groups compared with the control group (*p* < 0.05).

The immunostaining of Iba-1 showed comparable data between the groups treated with the pretreatment regimen, indicating that microglial activation was not increased in these four groups (Figure 6). However, the cytokine expression was different between the four groups. TNF-α was assessed in both the serum and hippocampal tissue extracts 6 h after the first dose of memantine and at two different time points after the end of paclitaxel treatment (Day 7 and Day 14). The high-dose memantine pretreatment suppressed TNF-α in both the serum and hippocampal tissue, as measured 6 h after the first dose of memantine, but this suppression did not persist throughout the course of the paclitaxel treatment. Serum TNF-α was elevated in the paclitaxel group (*p* < 0.01) and memantine + paclitaxel group (*p* < 0.01) at the end of the chemotherapy course compared with that measured in the control group (Figure 6). The TNF-α level in the hippocampal tissue on Day 7 was significantly elevated in the paclitaxel group (*p* < 0.05), and the reduction in the TNF-α levels in the hippocampal tissue was less obvious in both the memantine and memantine + paclitaxel groups. On Day 14, the TNF-α levels in the serum and hippocampal tissue were comparable between the groups. The expression of serum IL-β was increased in the paclitaxel group (*p* < 0.05) and memantine + paclitaxel group (*p* < 0.01) on Day 7, but the data regarding hippocampal IL-1β were relatively heterogeneous in the four groups and lacked a definite trend (Supplementary Figure S1).

### 3.3. Memantine Cotreatment Restored Hippocampal Neurogenesis, Improved Spatial Memory Deficits and Did Not Result in Depression-like Behavior

#### 3.3.1. Memantine Cotreatment Restored Hippocampal Neurogenesis

**Figure 7:**
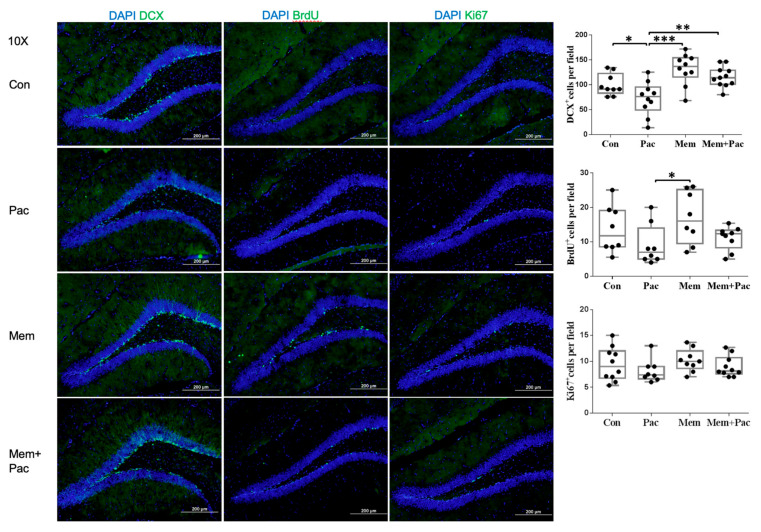
Cotreatment regimen (Experiment 3) (*N* = 8–10 in each group). Paclitaxel impaired neurogenesis, especially the neuroblast production in the dentate gyrus. The neuroblasts were marked as DCX-immunopositive cells. The BrdU- and Ki-67-immunopositive cell counts tended to be reduced in the paclitaxel group, but the differences were not statistically significant compared with the control group. The memantine pretreatment enhanced neurogenesis by increasing the Dcx- and BrdU-immunoreactive cell counts and restored neurogenesis by increasing the Dcx-immunopositive cell counts in the memantine + paclitaxel group compared with the paclitaxel group, but the Ki-67-immunopositive cell counts were similar between the groups. * *p* < 0.05, ** *p* < 0.01, and *** *p* < 0.001.

Experiment 3 aimed to test whether the cotreatment with memantine had better suppression of the inflammatory cytokines. Figure 7 shows representative images and the analyzed data of the Dcx-, BrdU-, and Ki-67-immunopositive cells. The Dcx-immunopositive cell count was still significantly increased in the group of mice treated with memantine compared with that in the paclitaxel group. The memantine cotreatment had a nonsignificant trend in increasing the BrdU-immunopositive cell count when compared with that in the paclitaxel treatment group. The Ki-67-immunoreactive cells were comparable between the groups.

#### 3.3.2. Memantine Cotreatment Improved Memory Deficits and Did Not Result in Depression-like Behavior

**Figure 8:**
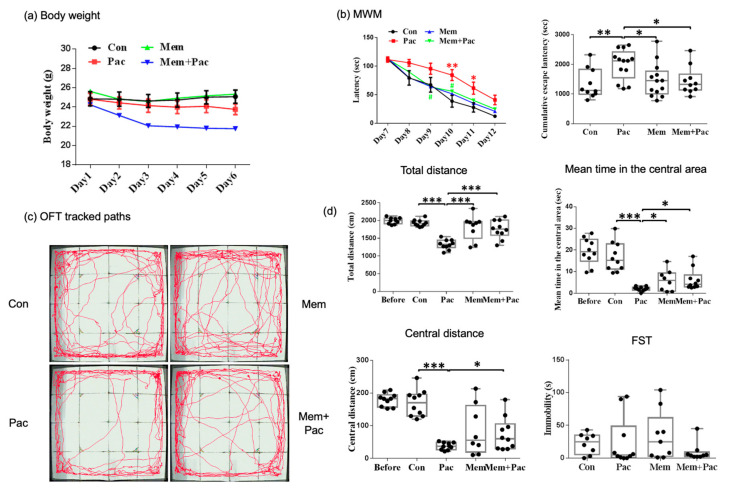
Cotreatment regimen (Experiment 3) (**a**) Body weights of the mice in the four groups after treatment. The data are presented as the mean ± SE of the mean. (**b**) The memantine cotreatment protocol significantly improved the spatial memory in the memantine + paclitaxel group. * Compared with the control group. # Compared with the paclitaxel group. (**c**) The OFT tracked the paths of the four groups. (**d**) Paclitaxel still impaired the locomotor activity in the paclitaxel group. However, the paclitaxel-treated mice cotreated with memantine exhibited a reversal of anxiety. The FST showed no prominent depression-like behavior in the memantine + paclitaxel group. Body weight: *N* = 5 in each group, MWM: *N* = 10 in each group, OFT: *N* = 10–15 in each group, and FST: *N* = 9–10 in each group. * *p* < 0.05, ** *p* < 0.01, and *** *p* < 0.001.

The body weights were slightly reduced in mice cotreated with memantine and paclitaxel, but the body weights were comparable among the other three groups (Figure 8a). The MWM test showed reduced overall escape latencies in the memantine + paclitaxel group compared with the paclitaxel group (*p* < 0.05, Figure 8b). The locomotor activity measured by the OFT was significantly increased in the memantine + paclitaxel group compared with the paclitaxel group (*p* < 0.001, Figure 8c,d). The distance and time in the central region were also significantly increased in the memantine + paclitaxel group compared with the paclitaxel group (*p* < 0.05, Figure 8c,d). The FST demonstrated less immobility time in the memantine + paclitaxel group (Figure 8d) than in the other three groups. This result indicated that the memantine cotreatment regimen may restore the spatial memory impairment and anxiety symptoms induced by paclitaxel and did not result in depression-like behavior.

#### 3.3.3. Memantine Cotreatment Significantly Increased Hippocampal BDNF Levels and Suppressed the Expression of Serum and Hippocampal TNF-α and IL-1β  in CICI Mice

**Figure 9:**
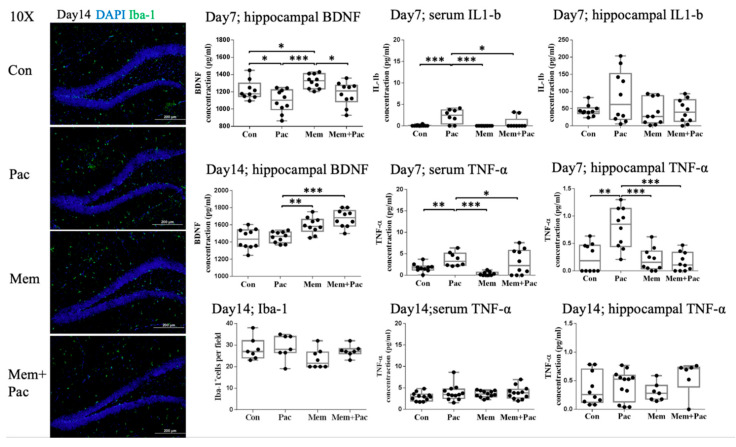
Cotreatment regimen (Experiment 3). The immunostaining for Iba-1 showed comparable microglial activation among the four groups. The serial expression of BDNF, TNF-α, and IL-1β in the four groups is also shown in Figure 9 and Supplementary Figure S2. The BDNF expression in the hippocampal tissue was increased after the memantine cotreatment on Day 7 and Day 14. After completion of the paclitaxel treatment, a reduced BDNF expression was noted in the paclitaxel group compared with the control group. Memantine suppressed the expression of TNF-α in the serum and hippocampal tissue on Day 7. The TNF-α expression in the serum and hippocampal tissue on Day 14 was comparable among the groups. Serum IL-1β  was also suppressed in the memantine-treated group. However, the tissue IL-1β  levels were heterogeneous in the groups. The serum and tissue IL-1β  data on Day 14 are shown in Supplementary Figure S2. Iba-1: *N* = 5 in each group, BDNF: *N* = 8–10 in each group, TNF-α: *N* = 8–12 in each group, and IL-1β: *N* = 9 to 10 in each group * *p* < 0.05, ** *p* < 0.01, and *** *p* < 0.001.

The ELISA analysis of the BDNF in the hippocampal tissue extracts was performed on Day 7 and Day 14. The results showed that the memantine cotreatment significantly increased the BDNF in memantine-treated mice on Day 7 and Day 14 compared with the paclitaxel treatment alone (Figure 9). Additionally, at the end of the chemotherapy course (Day 7), the BDNF was significantly reduced in the paclitaxel-treated mice compared with the control group (*p* < 0.05, Figure 9).

The immunostaining of Iba-1 showed comparable data between the groups receiving the cotreatment regimen, indicating that the microglial activation was not increased in these four groups (Figure 9). TNF-α and IL-1β were assessed in the serum and hippocampal tissue extracts on Day 7 and Day 14 (Figure 9 and Supplementary Figure S2). The memantine cotreatment significantly suppressed TNF-α in the serum and hippocampal tissue on Day 7, but the expression of TNF-α was comparable between the groups on Day 14. The memantine cotreatment also suppressed the serum expression of IL-1β (Figure 9), but the serum and hippocampal tissue expression of IL-1β was comparable between the groups on Day 14 (Supplementary Figure S2). These results indicated that the cotreatment regimen successfully suppressed the expression of proinflammatory cytokines in the serum and tissue, especially the expression of TNF-α, during the course of chemotherapy.

## 4. Discussion

Chemotherapy improves the long-term survival after a cancer diagnosis, but it may result in acute and/or delayed effects on the central or peripheral nervous systems [26,27], thus leading to a reduced quality of life for cancer survivors [28]. Many mechanisms have been proposed for CICI [3,28]. However, the exact mechanism is still not well-defined. Our study demonstrated a model of paclitaxel-induced CICI and investigated the potential relationship between CICI, impaired neurogenesis, and inflammation. In particular, we used memantine, a clinically available drug that has been reported to be a neurogenesis enhancer and inhibitor of neuroinflammation [23,29], as a potential therapeutic agent to modulate the paclitaxel-induced impairment of neurogenesis and inflammation. As such, experiments were designed to investigate the potential clinical treatment strategies for CICI.

Our data suggested that paclitaxel may induce impaired hippocampal neurogenesis and, thus, lead to spatial memory dysfunction and increased anxiety levels. The memantine treatment was shown to reverse memory symptoms and impaired neurogenesis through inducing an increased hippocampal BDNF expression and suppression of proinflammatory cytokines, and these two factors have both been reported to influence adult hippocampal neurogenesis [20,30]. However, using high-dose memantine as a pretreatment may not reverse anxiety and, further, may result in depression-like behavior, as demonstrated by our FST data. This finding may be explained by the inconsistent cytokine inhibition or a synergistic drug adverse effect. In the cotreatment model, giving memantine concomitantly during the paclitaxel treatment led to a potentially better suppression of peripheral TNF-α  and IL-1β  and tissue TNF-α  expression, thus leading to a better reversal of mood symptoms and prevention of depression-like behavior.

More than one specific mechanism of toxicity has been linked to chemotherapy-induced peripheral or central neuropathy, including CICI-related cognitive impairment and behavioral abnormalities [31]. One of the proposed mechanisms is the chemotherapeutic agent-induced blockade of hippocampal neurogenesis [4]. Cyclophosphamide and doxorubicin were related to a nearly 90% reduction in neurogenesis, and this reduction was associated with hippocampal-dependent memory impairment in an animal model [32]. Other reported chemotherapeutic agents that have a negative impact on hippocampal neurogenesis and neurocognitive functions include carmustine, cisplatin, temozolomide, cytosine arabinoside, methotrexate, and tamoxifen [4]. Paclitaxel is an effective chemotherapeutic agent against various types of cancers. It has been demonstrated to induce CICI [33,34]. Some mechanisms have been reported [7,35], including endoplasmic reticulum stress and the paclitaxel-induced impairment of microtubule dynamics. The relationship between paclitaxel-induced CICI and impaired neurogenesis has been less-discussed and could be related to the poor BBB penetration of paclitaxel. However, one report published by Lee. et al. in 2017 showed that paclitaxel may induce the downregulation of ventricular zinc, thus leading to impaired neurogenesis, especially neuroblast production labeled by Dcx immunostaining [8]. Our data further supported the relationship between paclitaxel-induced CICI and impaired hippocampal neurogenesis, again demonstrating an acute reduction in the differentiation of progenitor cells into neuroblasts but not proliferating cells labeled by BrdU and Ki-67 immunostaining. Notably, the dose of paclitaxel in our study comprised two doses, 6 mg/kg in Exp 1 and Exp 3 and 10 mg/kg in Exp 2, both for 6 consecutive days. The dose of paclitaxel in Lee’s report was 10 mg/kg for 7 days, and their results (a reduction of Dcx-positive cells, not BrdU- or Ki-67-positive cells) were similar to our data in Exp 1. They proposed that a paclitaxel dose of 10 mg/kg for 7 days may suppress microtubule dynamics and microtubule detachment from centrosomes, thus inhibiting cell division [8]. However, Atarod’s study in 2015 [25] showed that a paclitaxel dose of 6 mg/kg for 6 days may inhibit microtubule dynamicity and impair memory formation. Although Atarod et al. did not analyze the neurogenesis conditions in their study, their findings may support the potential inhibitory effect of microtubule dynamics in lower doses of paclitaxel. Therefore, in our study, paclitaxel at doses from 6 mg/kg to 10 mg/kg was tested to investigate the potential dose effect of paclitaxel on neurogenesis and inflammation. We found that both doses could lead to CICI symptoms and impaired neurogenesis. In contrast to the previous two reports, we further demonstrated that mood alterations should also be considered in the CICI model, since mood symptoms are common in patients with CICI.

Through our data, we noted that a paclitaxel injection may lead to reduced hippocampal BDNF and elevated peripheral and hippocampal proinflammatory cytokines, but acute microglial activation was not prominent at either dose of paclitaxel. The BDNF and the immune system both have regulatory effects on adult hippocampal neurogenesis [21]. Neurogenesis may be upregulated by the BDNF, and memantine was shown to increase the expression of BDNF in the brain [36]. The dose of memantine needed to enhance neurogenesis varied in previous studies. A single dose of memantine (35–50 mg/kg) may increase the number of BrdU-immunopositive cells 2 to 3 days after injection [23,37]. Therefore, the memantine dose in our two regimens was at least a total dose of 50 mg/kg. From our data, both memantine treatment protocols increased the BDNF expression but had varied modulating effects on the cytokine expression, especially TNF-α.  TNF-α  was shown to exert a negative impact on the survival of new neurons generated from neural stem cells in the subgranular zone and subventricular zone, especially through TNF-α receptor 1 [38]. In the pretreatment regimen, the cytokine-modulating effect was less effective than that in the cotreatment regimen, but the restoration of neurogenesis and corresponding spatial memory improvement were still noted in the pretreatment regimen. Thus, the effect of memantine on enhancing neurogenesis may occur through the regulatory effect of the BDNF.

Mood alteration is also one of the clinical symptoms of CICI. In our study, the mood changes after paclitaxel and different memantine regimens might help to explore the potential mechanisms of mood symptoms in the CICI model. Jacobs et al. in 2000 reported that impaired adult hippocampal neurogenesis may trigger depression [39] and, also, anxiety-related behaviors [40]. In our study, although neurogenesis was restored, the pretreatment regimen did not reverse the anxiety symptoms, and the combination of high-dose memantine and paclitaxel induced depression-like behavior. This is a surprising adverse synergistic effect. Our data showed that impaired neurogenesis and BDNF inhibition were successfully remedied with the pretreatment regimen; thus, persistent anxiety and depression-like behaviors may not be related to these two factors. We hypothesized that this may be related to the unsuccessful inhibition of peripheral and central TNF-α and peripheral IL-1β  in the pretreatment regimen. Previous studies have shown that TNF-α in plasma and cerebrospinal fluid was correlated with depression symptoms [41,42] and anxiety [43]. Peripheral inflammation, such as that from infection or tissue damage, can also lead to depression through immune-mediated pathways and transmit signals from the periphery to the CNS, for example, via the primary afferent nerves innervating the inflammatory site, via cytokines produced from the choroid plexus and circumventricular organs, and via specific saturable receptors [44]. Paclitaxel, a drug reported to have a lower BBB penetration, may induce the peripheral production of TNF-α, and peripheral TNF-α may reach the brain via the second and third pathways. The upregulation of TNF-α may stimulate the hypothalamic and pituitary axis (HPA), which has an important relationship with mood symptoms [45,46]. TNF-α  also activated neuronal serotonin transporters and stimulated indoleamine 2,3-dioxygenase (IDO), which may lead to tryptophan depletion, the reduction of serotonin, and the promotion of glutamate release [44]. The combination of the above TNF-α-associated neuropathological changes may thus result in persistent mood symptoms in the pretreatment regimen. In addition to TNF-α, IL-1β  is also implicated in the pathophysiology of mood alterations. Peripheral IL-1β  has been found to be increased in depressed patients [47] and was also associated with the onset of inflammation and chronic stress-related anxiety [48]. Paclitaxel was shown to enhance the innate immunity by promoting nucleotide-binding domain leucine-rich repeat (NLR) and pyrin domain containing receptor 3 (NLRP3) inflammasome activation in macrophages, and NLRP3 activated caspase-1, which cleaved pro-IL-1 into secretory cytokines [49]. In our study, the memantine pretreatment could not suppress paclitaxel-induced peripheral IL-1β  elevation well, thus potentially resulting in the persistent mood symptoms in our CICI model with the memantine pretreatment.

From our study, the sole paclitaxel treatment did not lead to obvious depression-like behavior, even under the higher dose of paclitaxel (10 mg/kg). Therefore, another proposed mechanism for the depression-like behavior in mice from combining high-dose memantine and paclitaxel was potential synergistic drug-adverse effects. Depression has been reported to be an uncommon side effect of memantine, although there is no exact incidence rate [50], and emotional distress may also be noted in paclitaxel-treated patients [51]. The previous literature has also shown that memantine cannot reverse hypomobility [52] and has no effect on depression [53]; as such, it does not have an acute antidepressant action. However, it may have an effect on reducing anxiety [53]. Thus, our data suggested that the mood symptoms of CICI could be reversed by the memantine treatment, but the effect of memantine on the mood may be influenced by the dose, treatment protocol, and the concomitant dose of the chemotherapeutic agents.

In our study, we adopted two different dosages of paclitaxel in the pretreatment and cotreatment regimens. In the pretreatment regimen, a poor inhibition of serum and tissue proinflammatory cytokine expression was noted after completing the administration of paclitaxel. Except for a potential poor inhibiting effect due to the pretreatment design of memantine, another potential reason for a higher cytokine expression may result from a higher dose of paclitaxel. However, the data from these two experimental designs may potentially explore the mechanistic interplay between the regimen designs of chemotherapeutic drugs and rescue drugs and the potential impact of inflammation and cytokine on the mood symptoms in CICI models.

Based on our results, memantine modulated neurogenesis, inhibited inflammation, and reversed the CICI symptoms. One previous clinical trial in humans also showed that add-on low-dose memantine may improve the plasma level of TNF-α and restore the clinical cognitive functions in bipolar II disorder [54,55], indicating a similar modulating effect of memantine on the inflammation and cognitive symptoms in different diseases. Our data might direct the potential possibility of considering memantine as an effective treatment strategy in CICI human studies. However, to translate these findings into clinical human studies, further investigations, specifically focusing on the doses and treatment regimens to avoid high doses and unnecessary pretreatments, may be warranted.

## 5. Conclusions

This study illustrates that impaired neurogenesis is one of the mechanisms underlying paclitaxel-induced CICI, and this impaired neurogenesis may be related to paclitaxel-induced BDNF inhibition and inflammatory cytokine induction. Memantine may be a potential effective treatment for CICI, but the treatment strategies may lead to variations in the treatment efficacy, especially in terms of the effect of mood alterations. Further translational studies may be developed to evaluate the clinical efficacy of memantine in human CICI studies.

## Data Availability

The data are available upon reasonable email request.

## Supplementary Materials

The following are available online at https://www.mdpi.com/article/10.3390/cancers13164177/s1: Figure S1: Pretreatment regimen (Experiment 2). The serum expression of IL-1β in the four groups. Figure S2: Cotreatment regimen (Experiment 3). The serum and tissue expression of IL-1β  in the four groups.
