# Supplementary material for: Memantine Protects against Paclitaxel-Induced Cognitive Impairment through Modulation of Neurogenesis and Inflammation in Mice
Source: Cancers (Basel). 2021 Aug 19;13(16):4177. doi: 10.3390/cancers13164177 (PMC8394018; doi:10.3390/cancers13164177)
Supplement: Supplementary file 1 [file cancers-13-04177-s001.zip › cancers-1325322-supplementary.pdf]

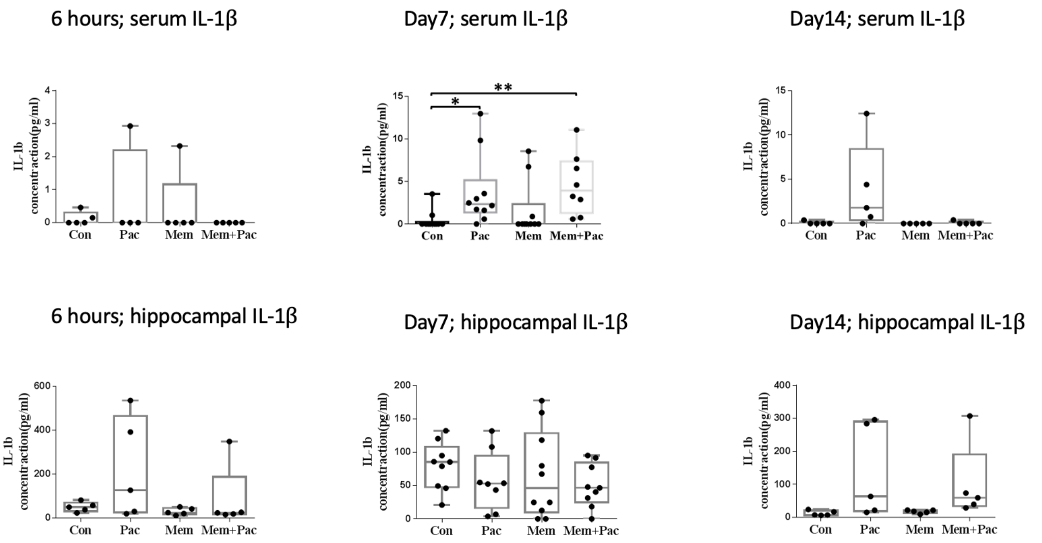

Supplementary Figure 1. Pretreatment regimen (Experiment 2). The serum expression of *IL-1β* was increased in the paclitaxel group and memantine + paclitaxel group on Day 7. However, the data was all comparable between groups in the hippocampal tissue at 6 hours, Day 7 and Day 14.

* $P<0.05$; ** $P<0.01$; *** $p<0.001$; **** $P<0.0001$; N= 5-10 in each group

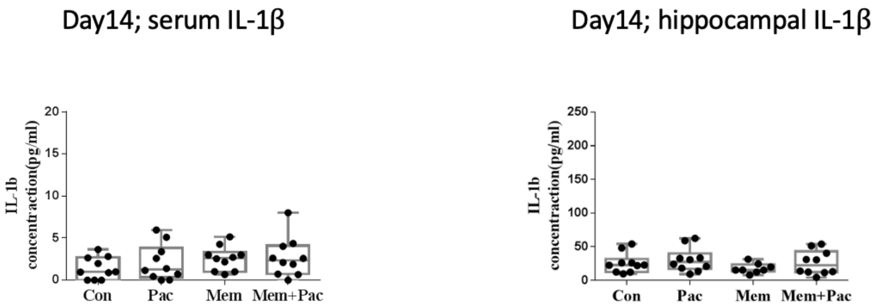

Supplementary figure 2. Cotreatment regimen (Experiment 3). The serum and hippocampal expression of *IL-1β* was comparable between groups on Day 14. N= 10 in each group
